# Supplementary material for: The effect of droplet size on syntrophic dynamics in droplet-enabled microbial co-cultivation
Source: PLoS One. 2022 Mar 31;17(3):e0266282. doi: 10.1371/journal.pone.0266282 (PMC8970485; doi:10.1371/journal.pone.0266282)
Supplement: S1 File — (DOCX) [file pone.0266282.s001.docx]

**Supplementary Information for**

The effect of droplet size on syntrophic dynamics in droplet-enabled microbial co-cultivation

James Y. Tan^1^, Tatyana Saleski^1^, Xiaoxia N. Lin^1*^

^1^Department of Chemical Engineering, University of Michigan, Ann Arbor

^*^Address correspondence to Xiaoxia Nina Lin, Department of Chemical Engineering, University of Michigan, G054W NCRC Bldg 28, 2800 Plymouth Rd., Ann Arbor, MI 48109-2800, USA

Email: [ninalin@umich.edu](mailto:ninalin@umich.edu)


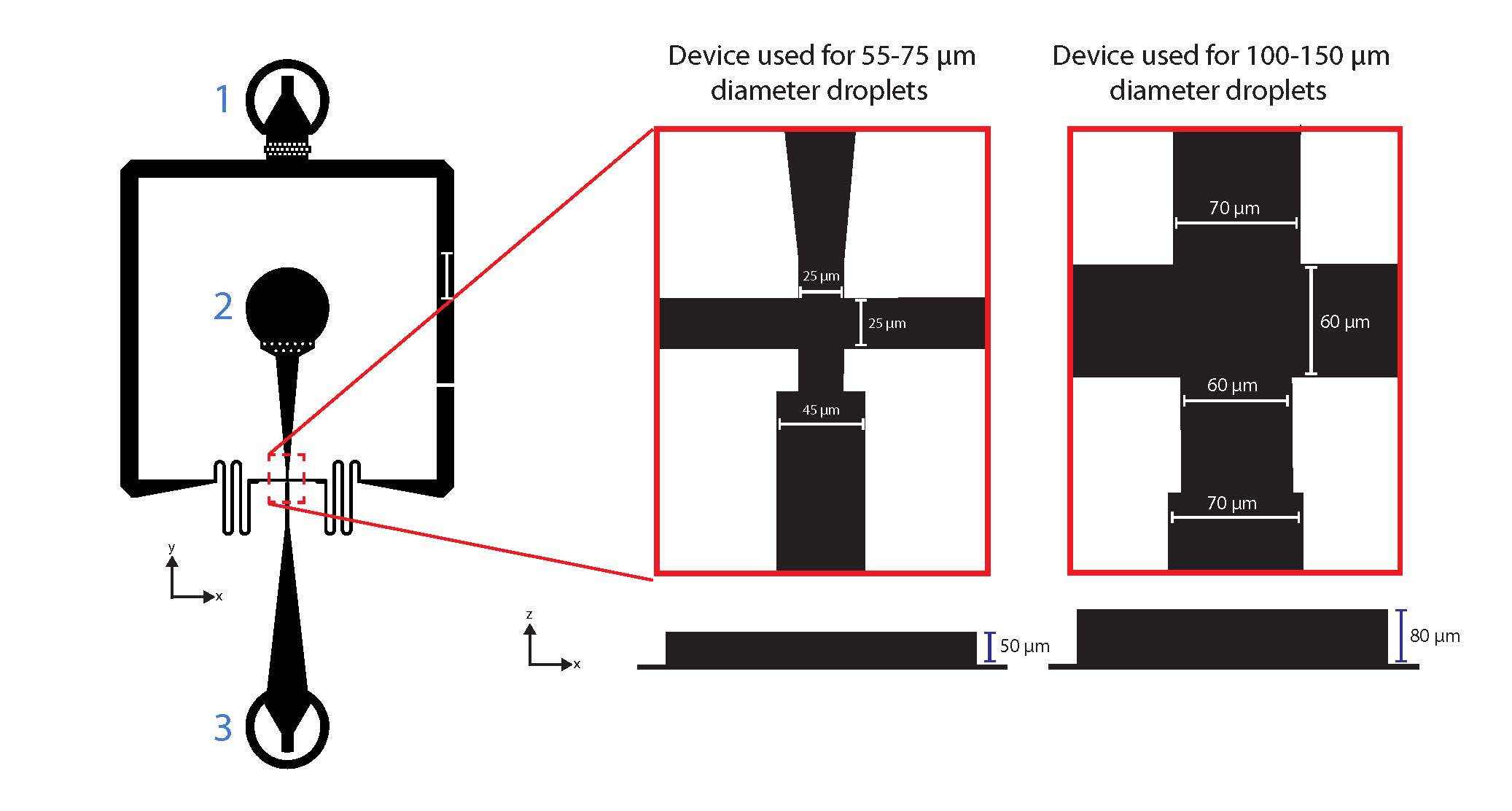


**Fig S1. Schematic layout of the SU-8 molds used to make the flow-focusing microfluidic PDMS devices used for droplet generation.** There were two devices utilized in the study which had similar design features but had different channel dimensions. All devices had an oil inlet (1), cell suspension inlet (2), flow-focusing channel intersection (boxed in red), and a droplet outlet (3). The first device mold (left) had channel widths of 25 μm and a channel height (blue scale bar) of 50 μm, while the second device mold (right) had channel widths of 60 or 70 μm and a channel height (blue scale bar) of 80 μm. The first device generated 55 μm and 75 μm diameter droplets, while the second device was used to generate 100, 125, and 150 μm diameter droplets.

**Note S1. Dynamic model of cross-feeding auxotrophs and parameter fitting with the logistic equation**

We observed that the growth profile of each auxotroph in the cross-feeding bi-culture exhibited the "S"-shape, characteristic of logistic growth commonly assumed for monocultures. To test, from a theoretical point of view, the validity of employing the logistic equation to approximate the growth of cross-feeding amino acid auxotrophs, we modified the mechanistic dynamic model by Kerner *et al.* (1) and carried out simulations to investigate growth dynamics resulting from interactions between two cross-feeding auxotrophs.

We assumed logistic growth for each auxotroph and the Monod equation relating the specific growth rate to the concentration of a limiting substrate (in this case the cross-fed molecule). The governing equations are as follows:

$$\frac{dn_{1}}{dt}=\mu_{1}n_{1}\left( 1-\frac{n_{1}}{K_{1}} \right)$$

$$\frac{dn_{2}}{dt}=\mu_{2}n_{2}\left( 1-\frac{n_{2}}{K_{2}} \right)$$

$$\frac{dc_{1}}{dt}=\alpha_{2}n_{2}-\beta_{1}\frac{dn_{1}}{dt}$$

$$\frac{dc_{2}}{dt}=\alpha_{1}n_{1}-\beta_{2}\frac{dn_{2}}{dt}$$

$$\mu_{1}=\frac{\mu_{1}^{max}c_{1}}{K_{s1}+c_{1}}$$

$$\mu_{2}=\frac{\mu_{2}^{max}c_{2}}{K_{s2}+c_{2}}$$

where dynamic variables $n_{1}$ and $n_{2}$ are the cell densities of the two auxotrophs, $c_{1}$ and $c_{2}$ are the concentrations of the cross-fed amino acids, and $\mu_{1}$ and $\mu_{2}$ are the (instantaneous) specific growth rates of the two auxotrophs. Model parameters $K_{1}$ and $K_{2}$ are the carrying capacities of the two strains following the logistic growth assumption, $\alpha_{1}$ and $\alpha_{2}$ represent the auxotrophs’ export rate of the amino acids, and $\beta_{1}$ and $\beta_{2}$ are their cellular requirement for the corresponding amino acids, respectively. $\mu_{1}^{max}$ and $\mu_{2}^{max}$ are the maximum specific growth rates of the two strains (i.e. at an infinitely high concentration of the limiting substrate); $K_{s1}$ and $K_{s2}$ are the “half-rate” constants in the Monod equation (i.e. concentration of the limiting substrate at which the growth rate is half of the maximum) for the two strains, respectively.

Using the above ordinary differential and algebraic equations with an initial condition of $c_{1}=c_{2}=0$ (i.e. no supplementation of the cross-fed molecules at the start), we carried out dynamic simulations to explore various scenarios representing different degrees of interactions between the two cross-feeding auxotrophs. Specifically, one of the $\beta$ parameters was changed to represent different levels of demand for the cross-fed metabolite. Our simulation results, as illustrated in Fig S2, demonstrate that the growth profile of each auxotroph in the cross-feeding bi-culture follows a general S-shape and can be approximated reasonably well with a logistic fit (R^2^ value higher than 0.98). The fit is particularly strong at the lowest degree of cross-feeding (i.e. when β is set at the smallest value).


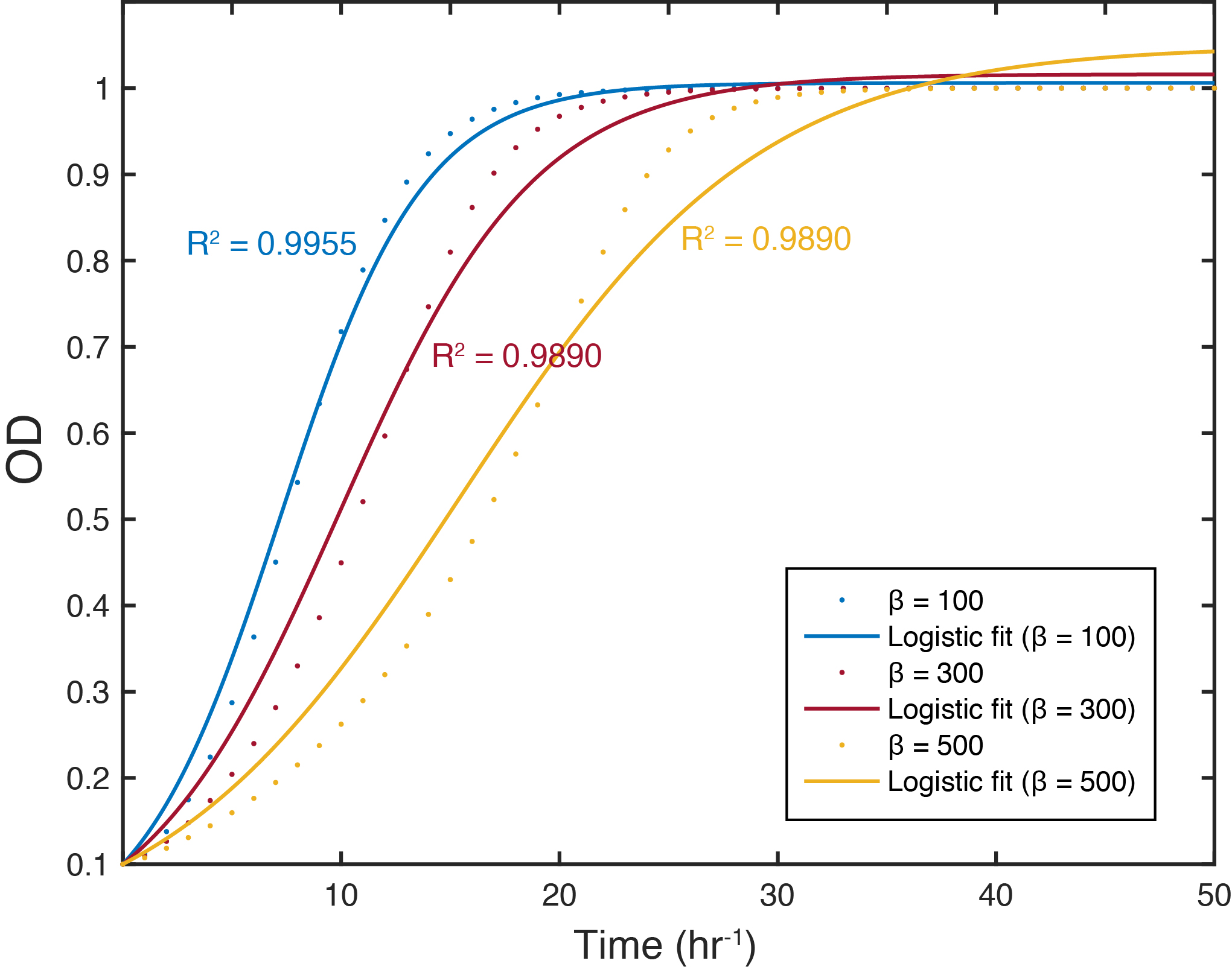


**Fig S2.** **Comparison between simulated growth dynamics of an auxotroph participating in mutual cross-feeding with another auxotroph and the profiles fitted using the logistic equation.** The cellular requirement for the cross-fed metabolite is represented by β and was adjusted from 100 to 300 and lastly to 500 μg/L-OD while all other parameters of the model were kept constant. The simulated growth curves of the auxotroph are plotted in dotted lines, while the respective logistic fits are provided in solid ones. The R^2^ value indicative of the quality of the fit is also provided for each fit.


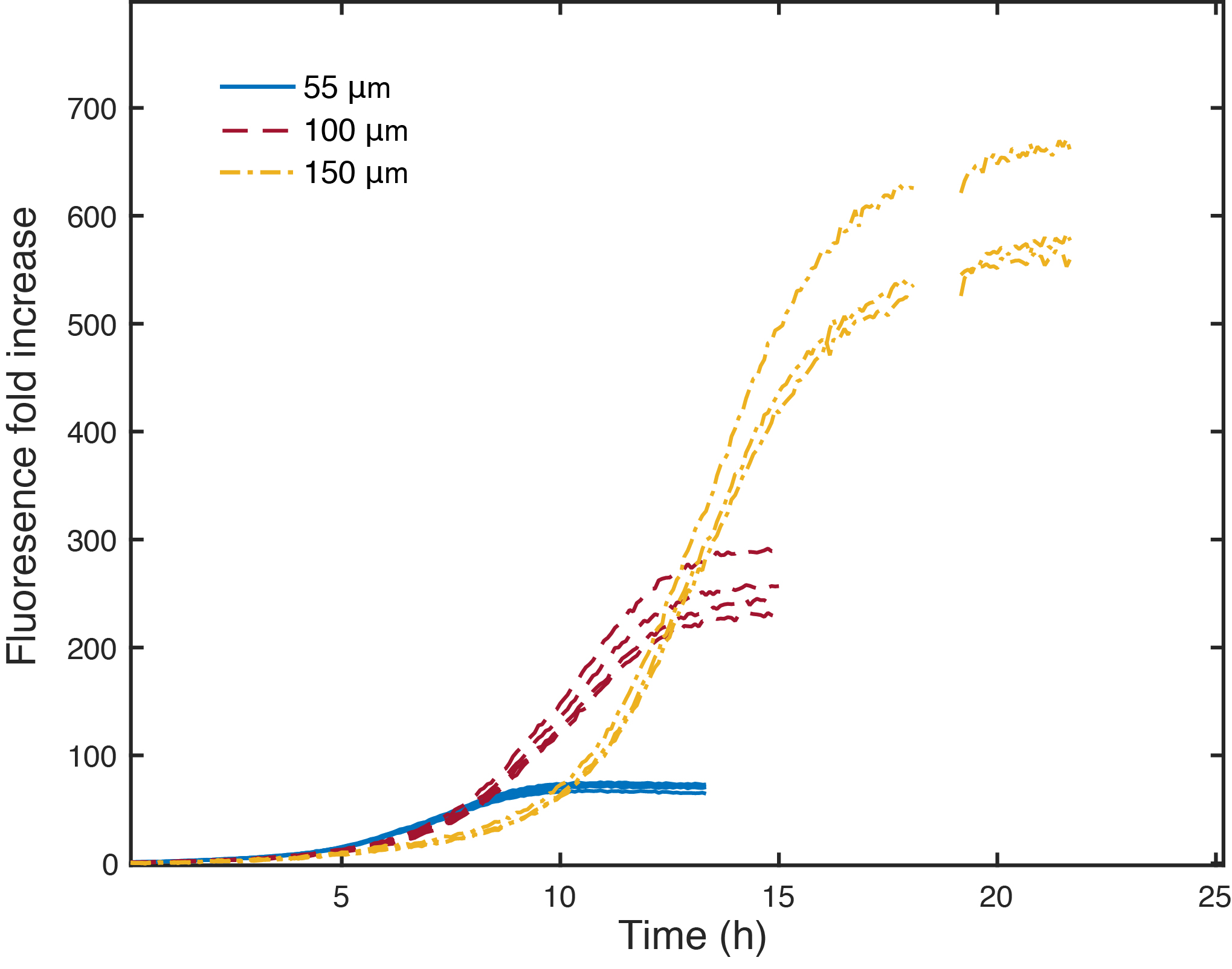


**Fig S3.** **The full set of growth curves for the monoculture microdroplet cultivation of S1 Δ*ilvD.*** The initial λ was 5 cells/droplet. Each curve is the aggregate growth of droplets within a single well in a 96-well plate, representing a large population of droplets. Fluorescence fold increase is the fluorescence at a time point normalized by the initial fluorescence of the sample well. Each condition had 4 replicates, with 3 for 150 μm diameter droplets due to one replicate having inaccurate initial measurements.


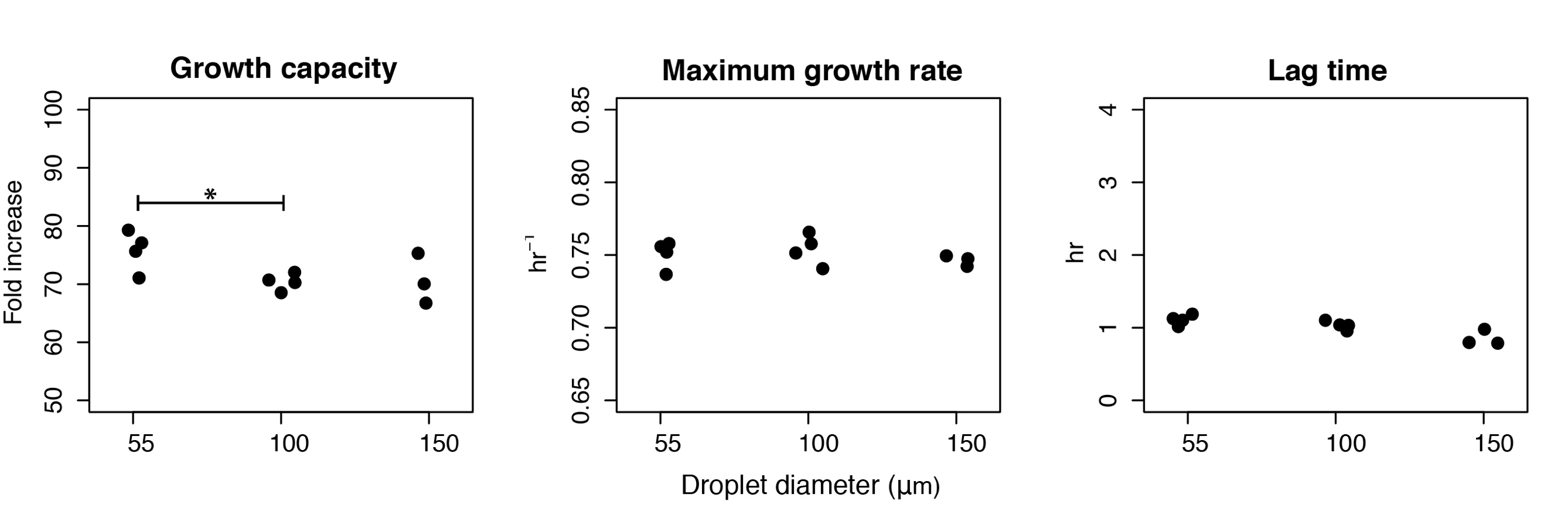


**Fig S4. Growth model parameters estimated from fluorescence data of S1 Δ*ilvD* monoculture grown with the same initial cell density in droplets of different sizes.** The average initial cell number (i.e. λ parameter of the Poisson distribution) was 5, 30, and 100 cells/droplet in 55, 100, and 150 μm diameter droplets, respectively. * indicates p-value < 0.05 for statistical significance.


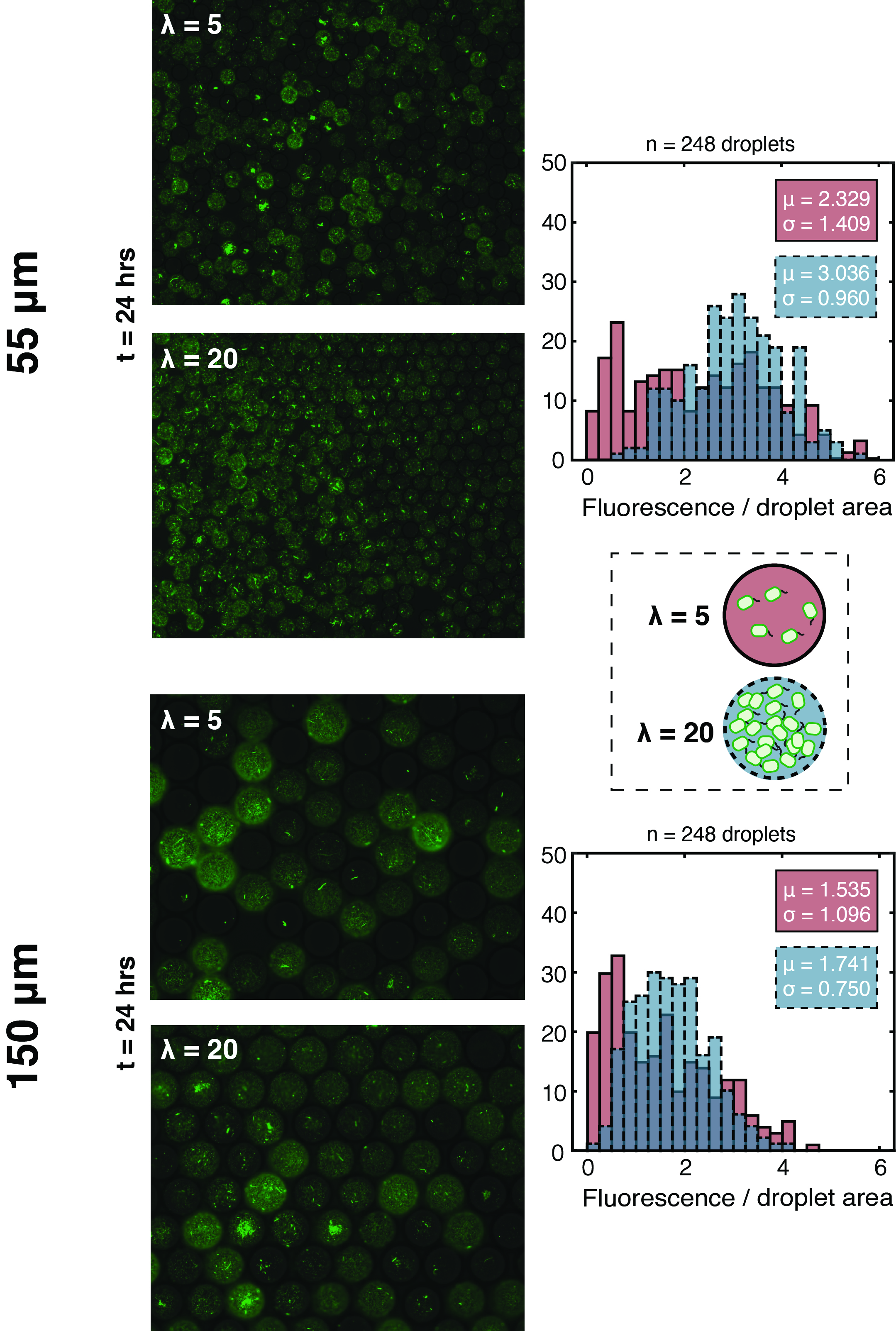


**Fig S5.** Droplet-to-droplet variance of fluorescence for the cultivation of S1 Δ*ilvD* with initial λ=5 and λ=20 cells/droplet in droplets with diameters of 55 and 150 μm. Droplet-to-droplet variation is observed in representative images of populations of droplets after cultivation for 24 hours under both conditions. Image analysis of a large population of droplets (248 droplets for each) was performed to quantify the degree of droplet-to-droplet fluorescence variation through histograms with associated statistics (mean and standard deviation). The distribution and statistics of the λ=5 cells/droplet condition is magenta with a solid boundary. The distribution and statistics of the λ=20 cells/droplet condition is blue with a dashed boundary.


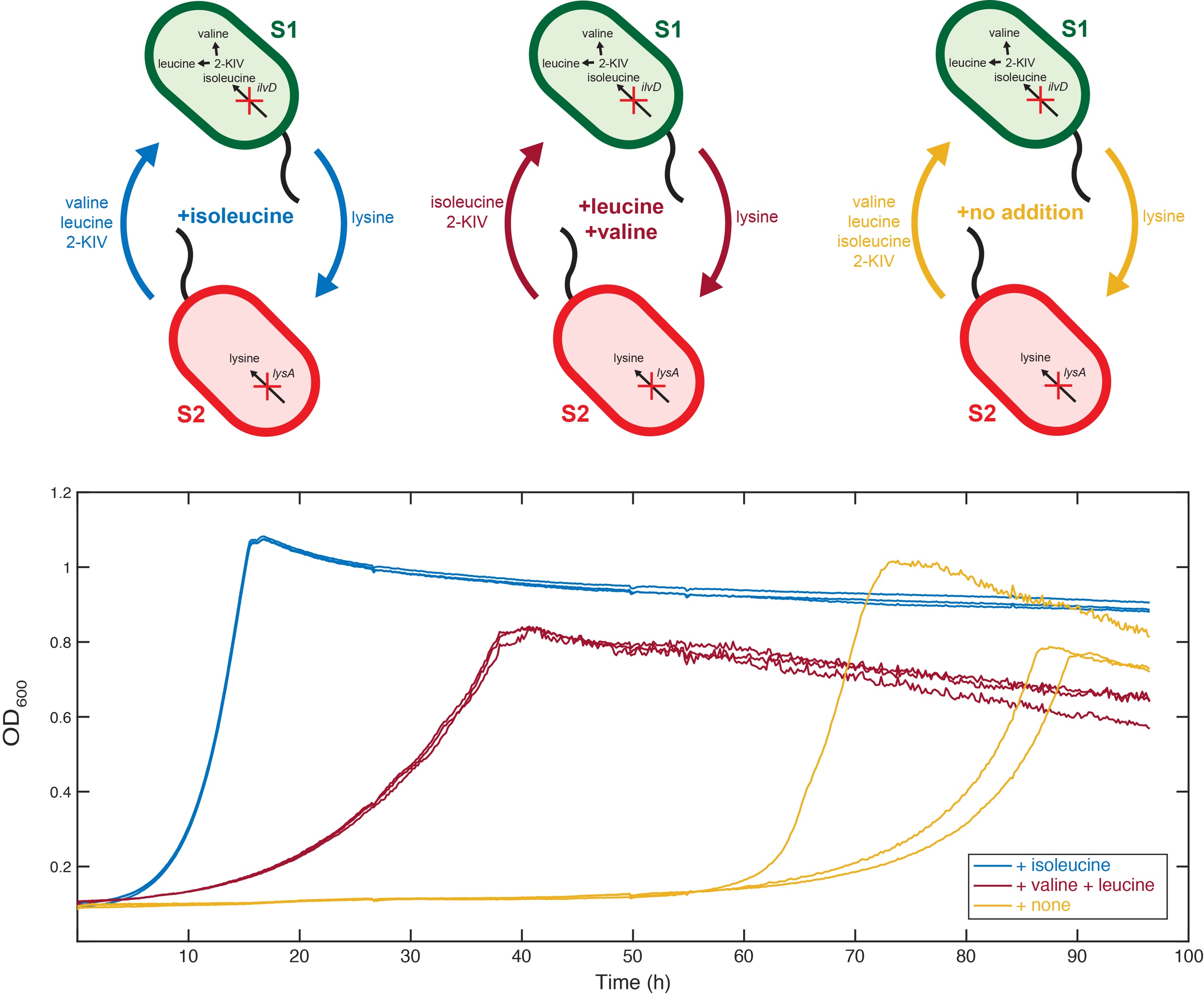


**Fig S6. Growth of co-cultures of S1 Δ*ilvD* and S2 Δ*lysA* in bulk.** Cultivation was done in microwell plates under three different amino acid supplementation conditions to modulate the degree of interaction between the two auxotrophic partners: (1) with 3 mM isoleucine, (2) with 3 mM leucine and 3 mM valine, and (3) with no addition of amino acids. Each condition had three replicates.


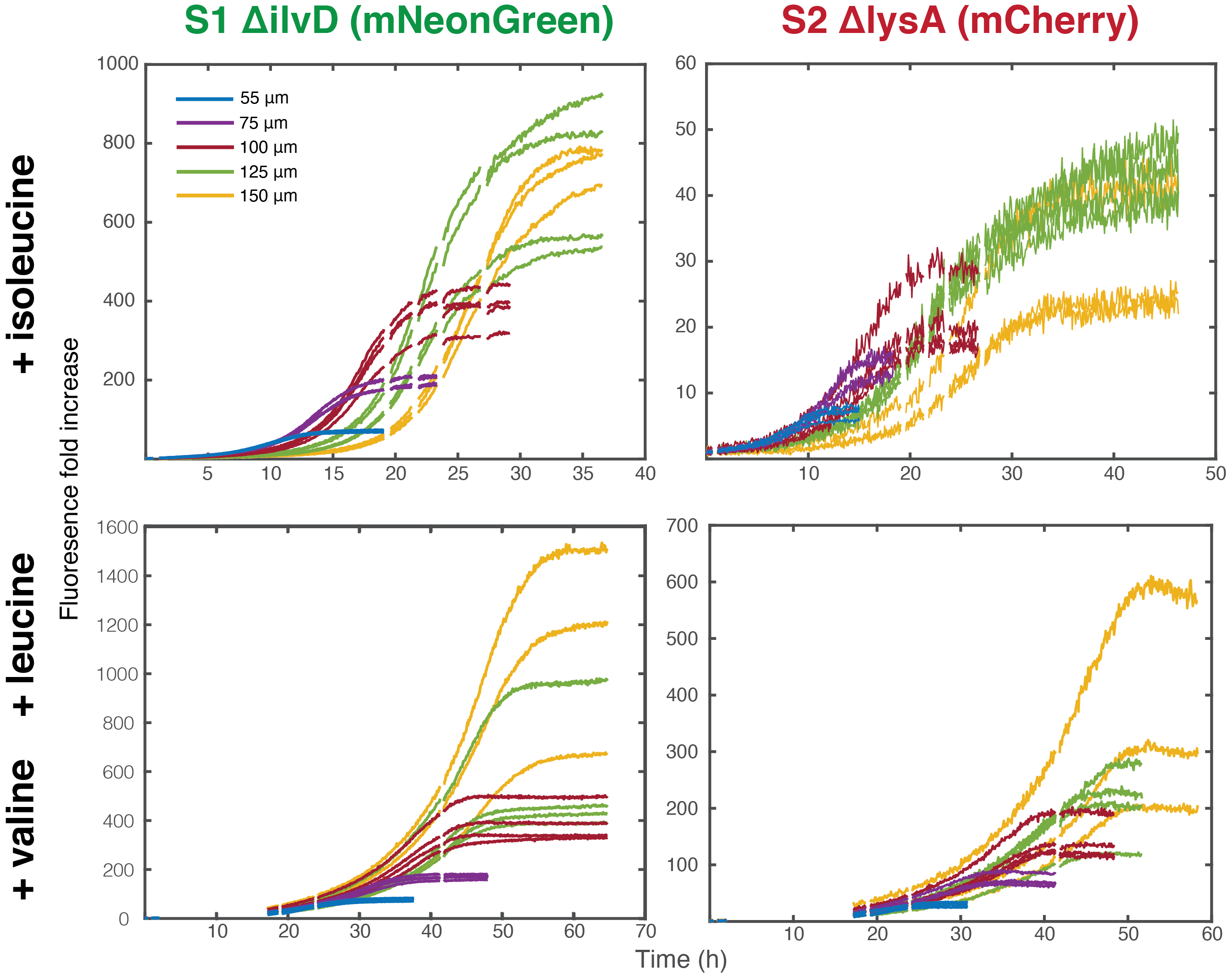


**Fig S7. The full set of growth curves from co-cultivation of S1 Δ*ilvD* and S2 Δ*lysA* in droplets**. The initial λ value was 5 cells/droplet of each strain under the two amino acid supplementation conditions. Each curve is the aggregate growth of droplets within a single well in a 96-well plate, representing a large population of droplets. Fluorescence fold increase is the fluorescence at a time point normalized by the initial fluorescence in the same well. Each condition had 4 replicates, with 3 for 150 μm diameter droplets due to one replicate having inaccurate initial measurements.


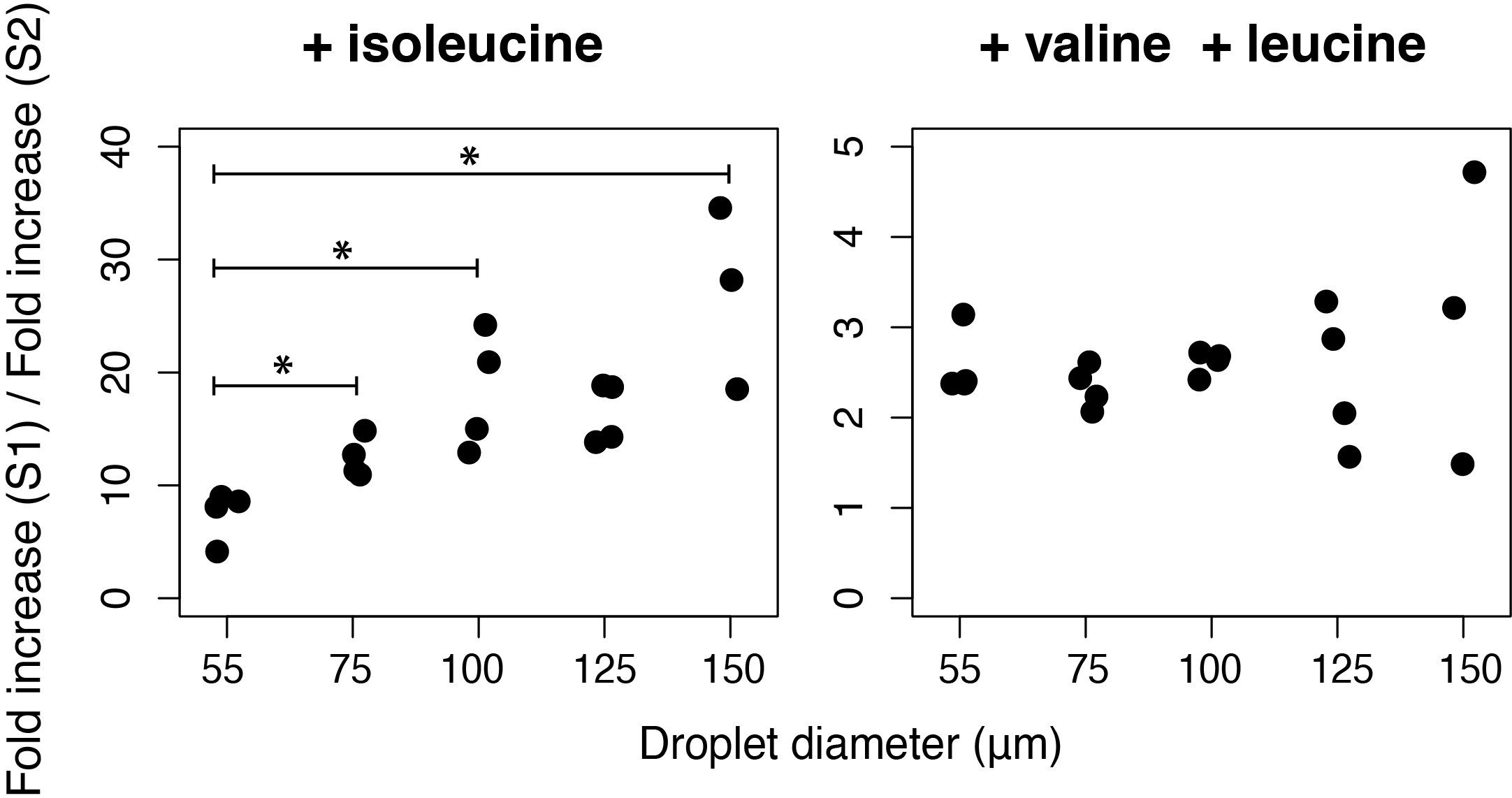


**Fig S8. The effect of droplet size on community composition between S1 Δ*ilvD* and S2 Δ*lysA.*** Community composition is provided as the ratio between fold increases of S1 Δ*ilvD* and S2 Δ*lysA* in droplets of different sizes when 3 mM isoleucine or 3 mM valine and 3 mM leucine is supplemented. For each well in the co-cultivation experiments, a ratio between the fold increase of S1 and that of S2 was calculated. Statistical significance is defined by a p-value of less than 0.05 (*).


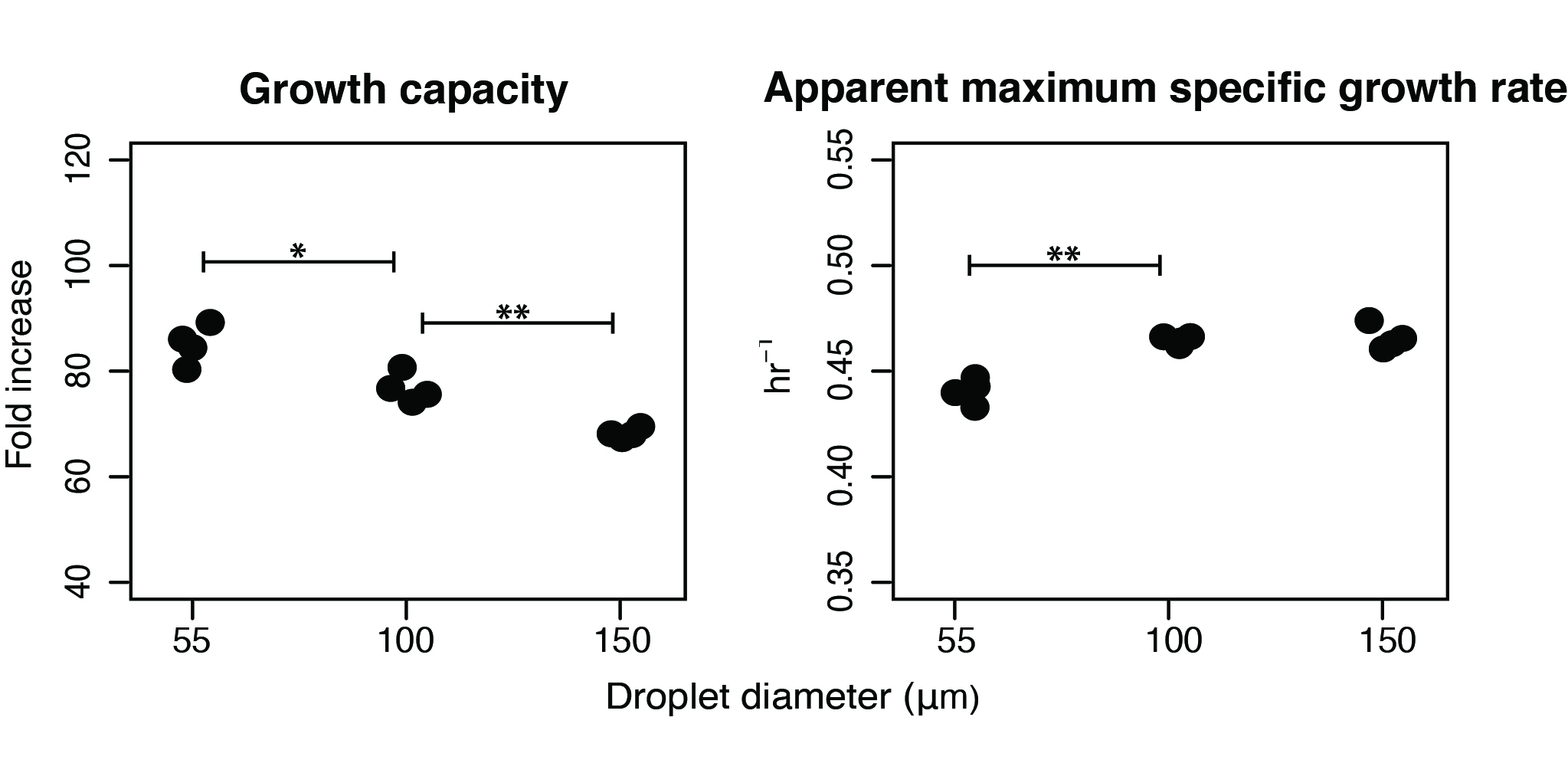


**Fig S9. Growth model parameters estimated from fluorescence data of S1 Δ*ilvD* grown in co-culture with S2 Δ*lysA* with the same initial cell density in droplets of different sizes.** The average initial cell number (i.e. λ parameter of the Poisson distribution) was 5, 30, and 100 cells/droplet for each strain in 55, 100, and 150 μm diameter droplets, respectively. Lag time was not evaluated due to there being an insignificant lag time observed. Statistical significance is defined by a p-value of less than 0.05 (*) and a p-value of less than 0.01 (**).


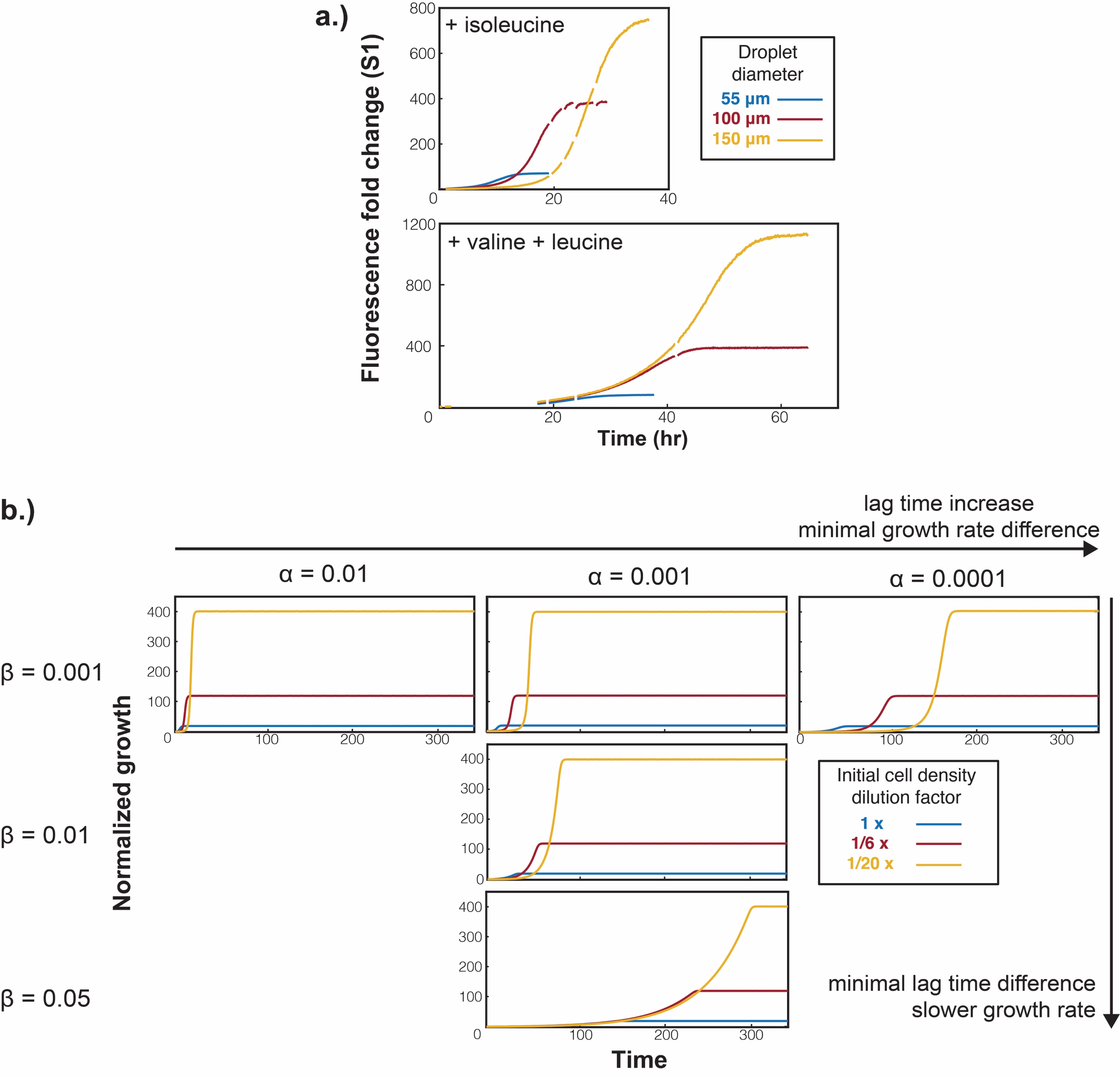


**Fig S10. Different parameter values of a mathematical model lead to different patterns of growth dynamics, recapitulating in part experimental observations.** (a) Experimental data for S1 Δ*ilvD* in co-culture with S2 Δ*lysA* with supplementation of isoleucine and valine/leucine (from Fig 3c and Fig 4c, respectively). Under the isoleucine supplemented condition, lag time is extended as droplet size increases. Under the valine and leucine supplemented condition, lag time remains largely the same and the growth curves overlap during the earliest phase. (b) Growth curves generated from the cross-feeding autotroph ODE model, with different parameter values for amino acid secretion (α) and cellular requirement of the other amino acid for growth (β). For each scenario, three sets of initial conditions were specified corresponding to changes of the initial cell density when the droplet diameter was increased from 55 to 100, and then to 150 μm. It was noted that the growth dynamics in the scenario of α = 0.0001 and β = 0.001 (top right sub-plot) exhibited qualitatively similar pattens to those in experimental profiles under the isoleucine supplemented condition (top sub-plot in a.), whereas the simulated growth profiles in the scenario of α = 0.001 and β = 0.05 (bottom center sub-plot) greatly resemble those in experimental profiles under the valine and leucine supplemented condition (bottom sub-plot in a.). Other parameters of the model were set as: μ­_max_ = 1, K_s_ = 0.1, and K = 100.

**Supplementary References**

1. Kerner A, Park J, Williams A, Lin XN. A Programmable *Escherichia coli* Consortium via Tunable Symbiosis. PLoS One. 2012;7(3):e34032.
