# Supplementary material for: The effect of droplet size on syntrophic dynamics in droplet-enabled microbial co-cultivation
Source: PLoS One. 2022 Mar 31;17(3):e0266282. doi: 10.1371/journal.pone.0266282 (PMC8970485; doi:10.1371/journal.pone.0266282)
Supplement: Attachment — Submitted filename: Tan_etal_Rebuttal_PLOSONE_v3.docx [file pone.0266282.s002.docx]

**Point-by-point response to the reviewer’s comments**

Dear Dr. Orman,

Thank you for the decision on our manuscript. We are encouraged by the positive reviews and the affirmation that this study was enlightening and important as the field continues to grow. The comments by the reviewers were also very helpful in pushing us to rethink some of our key conclusions regarding the role of diffusion. We have revisited the relevant sections of our manuscripts and made very substantial revisions. In particular, we have conducted an additional set of experiments and new modeling work, based on which we incorporated four new figures (Fig. 2 in the main text and Figs. S5, S9, S10 in the supplement) and re-wrote a significant part of the text, particularly in Discussion. These revisions are described below in our specific responses to the reviewers’ comments and in a summarizing list.

Thank you for considering our revision.

Sincerely,

James Y. Tan (First author)

Xiaoxia Nina Lin (Corresponding author)

**Reviewer #1**

***Comment:*** *The interpretation of the results that growth dynamics vs droplet size is caused by diffusion I think is plausible, but the authors need to describe this interpretation in more detail. What are the diffusion rates and timescales for the relevant metabolites? Also, how does this compare to the fact that the E coli are highly mobile? As the E coli are swimming around, don’t they leave a wake of metabolites? Numerical order of magnitude estimations are needed here which describe all relevant scales -- diffusion, E coli mobility, mass transport.*

**Response:** We appreciate this very thoughtful comment. As suggested, we carried out an order of magnitude estimation for the time scale of diffusion in microdroplets, and the results were very helpful in allowing us to re-evaluate our interpretation of experimental observations. Specifically, we estimated the average time required for a representative amino acid molecule to travel the longest distance in a droplet based on statistical mechanics and recognized that the time scale involved in molecular diffusion is much shorter than that associated with cell growth. Hence, diffusion is not likely a major contributing factor underlying our experimental results. We have included these calculations and discussions throughout the revised manuscript (in Introduction, Results and Discussion).

Regarding cell mobility, *E. coli* is indeed motile and can move on the scale of microns/sec. If *E. coli* is moving while secreting, this will also contribute to the secreted metabolites being spread in the droplet and then quickly diffused. However, as discussed above, we now speculate that transport of the secreted metabolites within the droplet is most likely not the limiting factor.

***Comment:*** *Growing microbes in droplets is a fast moving field -- which makes the authors publication very relevant -- but the authors also need to update their references to cover more recent droplet investigations of bacteria growth. A starting list is:*

- *Villa, Max M., et al. "Interindividual variation in dietary carbohydrate metabolism by gut Bacteria revealed with droplet microfluidic culture." Msystems 5.3 (2020): e00864-19.*
- *Mahler, Lisa, et al. "Highly parallelized droplet cultivation and prioritization of antibiotic producers from natural microbial communities." Elife 10 (2021): e64774.*
- *Watterson, William J., et al. "Droplet-based high-throughput cultivation for accurate screening of antibiotic resistant gut microbes." Elife 9 (2020): e56998.*

**Response:** The field truly does move fast and we appreciate the reviewer’s thoughtful suggestion! These more recent references, along with several others, have been added in the introduction.

**Reviewer #2**

***Comment:*** *(important point) Line 56-57: The authors claim that droplets are increasingly used to study interactions. I think this is true and that what has been published so far is only the early beginning of many such methods to be developed and used. But the references 1-3 do not involve droplets, and the following references provide an incomplete overview mainly based onolder publications, not reflecting the increment. I suggest a few relevant and incomplete recent examples from third partyresearch groups that I think help show this trend and its relevance: DOIs: 10.1073/pnas.1811250115;10.1039/D0LC01204A;*

*10.1016/j.copbio.2019.09.001; 10.7554/elife.64774 (just individual strains)*

**Response:** We thank the reviewer for looking very carefully at the reference list in our original submission and providing these excellent suggestions. We have removed the wrong references and added the suggested references, along with several others, in the first paragraph.

***Comment:*** *Line 85: “In bulk culture, convective mass transfer is ...seldom a limiting factor...". This sounds a lot like gut microbiome, but I don't think it generally holds for other habitats such as soil, tissues, or even ocean zones. Please correct.*

**Response:** The original intention was to describe laboratory flask cultures which are typically well-mixed, but the reviewer brings up a good point to put this in perspective with natural systems where there is a continuum of homogeneity and heterogeneity. We have revised this part as suggested and expanded our discussion.

***Comment:*** *Line 111:* *A wide range of droplet sizes can be made with the same device, especially when switching between jetting and dripping by changing flow ratios and speeds. The current sentence sounds like this is not possible and I recommend an adjustment.*

**Response:** We agree. This study itself uses two droplet devices to generate 5 different sizes in the range of 55 to 150 μm. This sentence has been removed.

***Comment:*** *(important) Line 415 and after:* *The results section contains large amounts of interpretation, which I would normally expect in the discussion section. In particular, in my view, the claim that diffusion of metabolites is the underlying mechanism of the observed variable changes in different droplet sizes is not supported by the data. It is a reasonable hypothesis to explain the trends, but as the authors point out in other parts of the manuscript, other parameters such as quorum sensing also influence the growth behaviour. I could not follow the conclusion that the diffusion mechanism is the sole reason, for this I would expect to see more molecular-level evidence. However, in my view this makes the study no less interesting. It still shows the droplet size dependence of an example microbial co-culture, with all its complex dependencies.*

**Response:** This is an excellent comment. This feedback and a similar one from another reviewer have pushed us to re-think about our previous interpretation. We have also conducted new investigations in an attempt to dissect more thoroughly the mechanisms underlying our experimental observations. In particular, using an ODE model we have previously developed, we examined how the growth dynamics change when key parameter values are varied and have been able to recapitulate part of the complex trends observed experimentally. These new findings suggest that in addition to or even instead of diffusion, inherent properties related to intercellular interactions in complex ecosystems give rise to specific trends of growth dynamics. Please see details in the new first paragraph in the “Discussion” section and a new figure in the supplement (Fig. S10).

***Comment:*** *Line 490: The droplet-to-droplet variation is an interesting side story of the article that I would ideally like to see explored in more detail as a reader (beyond the short discussion from line 549 onwards), as it is equally relevant for interaction study design in droplets. To what degree is this variation explained by the stochasticity of the poisson distribution at lambda = 5? Also, it would be great to see more justification or explanation around this value "5" as a choice for almost all experiments presented. How much can be assigned to secretion, and how much to fluorescent protein generation? Are there other systematic studies to give partial answers? And in particular for interactions: What is the relevance of the stochastic co-encapsulation? For example if one partner is encapsulated at higher cell numbers as the other by chance (or systematically by varying the lambda value for one, but not the other strain).*

*Related to this, please clarify the sentence in line 520: Does the variability only depend on the limited growth capacity in smaller droplets? How did you reach that conclusion?*

**Response:** These are all very insightful questions. We have carried out an additional experiment to determine if an increase in the initial lambda value would decrease the amount of droplet-to-droplet variability. We investigated this hypothesis in a simple monoculture experiment and added two new figures (Fig. 2 and Fig. S5 in the revised manuscript) to demonstrate that variability can be decreased by increasing the initial lambda.

We have clarified the choice of lambda = 5 in the text, that it was to keep the initial cell number per droplet low while trying to ensure that almost every droplet had at least one cell.

It is difficult to fully assess how much the variability is assigned to each of these components (Poisson distribution, cell viability, growth/secretion, and fluorescent protein generation). The added experiment with higher initial lambda demonstrates that even with variation introduced from the Poisson distribution, the variability is decreased with higher lambda. This result suggests that the cell-to-cell variability is what is contributing the most to the droplet-to-droplet variability observed at low lambda values.

It would be interesting to try systematically varying the lambda value of one strain and keeping the other constant. Extrapolating from our experiment where we increased the lambda, we would expect the characteristics of the one strain at a higher lambda to be more consistent across droplets, whereas the lower lambda strain would be expected to exhibit a higher degree of droplet-to-droplet variability. This could be investigated in future studies.

The statement about variable community composition due to limited growth capacity was confusingly worded. It was a statement on the change of community composition across different droplet sizes for the same condition, not on droplet-to-droplet variability. The statement has been removed.

***Comment:*** *On data availability: All the used code seems to have been made available in detail, including scripts to generate the figures, but I did not see the raw data.*

**Response:** We appreciate the reviewer’s attention to detail. The raw data was not included in our original submission, but the Github repository has the raw data and description of how the raw data was converted to normalized data required for the MATLAB scripts.

**Summary of Changes**

Revisions are listed by section with respect to the original manuscript and are noted in terms of either the section sub-header or the paragraph number.

*Abstract:*

- Included both diffusion and dilution as potential mechanisms for the impact of droplet size on community dynamics
- Removed conclusion that effects are due to diffusion

*Introduction:*

*Paragraph 1:*

- Included updated references recommended by reviewers

*Paragraph 3:*

- Updated the discussion to explore the spectrum of mass transfer in natural environments
- Included some order-of-magnitude estimations of time required to diffuse a distance in 3D

*Paragraphs 5 and 6 (now Paragraph 4):*

- Collapsed these paragraphs into one paragraph, discussing the parameters to consider in droplet encapsulation
- “However, if the objective is co-culture encapsulation…” -> “However, if the objective is co-culturing…”
- Removed comment on microdroplet dimensions being relatively fixed in droplet generation devices.
- “As a result, droplet size is usually…” -> “As a result, droplet can be…”
- “…is not a trivial parameter to determine either.” -> “…is not a trivial parameter to determine.”
- “This is highly dependent” -> “This can be highly dependent”
- “…the diffusivity of the metabolite…” -> “…the diffusivity and affinity of the metabolite…”

*Paragraph 7 (now Paragraph 5)*

- “Metabolite exchanges” -> “We hypothesized that metabolite…”

*Materials and Methods:*

Growth kinetics analysis

- *Paragraph 1:*
  - Included that “the background fluorescence was removed from growth data and were normalized…”
- *Paragraph 2:*
  - Symbols were changed to symbols instead of equation editor text as per PLOS ONE specifications
  - “The raw and normalized data” are both included in the Github
- *Paragraph 3*:
  - Included the citation for what model we built off of
  - Added a comment about altering the model to study the effect of increased droplet size by decreasing the initial cellular density

*Results:*

The growth dynamics of a monoculture as a baseline for understanding droplet-enabled cultivation

- *Paragraph 1:*
  - Added a statement clarifying the choice of lambda = 5
- *Paragraph 2:*
  - “um” changed to “μm”
- *Paragraph 3:*
  - Changed wording from “unobvious” to “unintuitive”
- *Paragraph 4:*
  - Changed “To gain further insight on how the droplet size impacts…” to “To control for initial cell density”
  - Included mention of what changing the droplet size could affect, such as quorum sensing or the diffusion of exchanged molecules
  - Removed text talking about how the finding suggest the importance of diffusion
- *Paragraph 5 (added):*
  - New discussion about new experiment increasing initial lambda and the effect on droplet-to-droplet variability
  - New figure included (Fig 2) and new supplementary figure (Fig S5)

A model system of cross-feeding amino acid auxotrophs

- Added statement clarifying choice of lambda of 5

Syntrophic growth in droplets is slowed down at larger droplet sizes

- Deleted discussion of diffusion being the contributing factor for observed phenomenon.
- Included a discussion of the new supplementary figure S9 which discusses an experiment where the initial cellular density was kept constant across droplet size, but for a co-culture with isoleucine supplementation.

With an increased degree of interaction, syntrophic growth is impacted even more substantially by the droplet size

- Updated Fig 4 to incorporate correct data. There was a background-removal error in the data used to make the previous figure, but these changes did not change results substantially.
- *Paragraph 2:*
  - “100 to 125 um droplets” -> “100 to 125 um diameter droplets”
  - “observed gradual” -> “observed a gradual”
  - Included approximate percent decrease in growth rate observed in Fig 4
  - Removed explanation of trends by diffusion
  - Lag time trends were no longer evident with the updated figure and reflected in observations.
  - Due to changed numbers in Fig S8 from previous background removal error, the finding was that community composition remained the same as droplet size increased, contrary to what was written previously. Rationale for why this is the case included.

With the highest degree of interaction, syntrophic growth can be substantially hindered in larger droplets to the extent where a significant subpopulation fails to establish co-growth

- Removed discussion of plasmid-associated variability to “Discussion”

*Discussion:*

- Entire section largely rewritten to include discussion of alternative mechanisms besides diffusion
  - New Fig S10 referenced to present preliminary data
- Removed discussion of diffusion being the expected mechanism
- Discussion of droplet-to-droplet variability included for the monoculture and co-culture
- Discussion of cell-to-cell variability included, such as plasmid contributions
- More detailed discussion of how the implications found in our study affect specific scenarios that microdroplet co-cultivation is used for.

*Acknowledgements:*

- Included an acknowledgement of the reviewers’ contributions by expressing new perspectives that helped us consider what mechanisms are explaining the results we observed.

*Supplementary Information:*

- *Fig S4:*
  - “…parameters extracted from microdroplet cultivation of S1 Δ*ilvD*” changed to “…parameters estimated from fluorescence data of S1 Δ*ilvD* monoculture grown…”
  - Lambda values in Fig S4 added.
- New supplementary figure added (Fig S5)
- Fig S7 updated to reflect the changes introduced by fixing the error in Fig 4.
- Fig S8 updated to reflect the changes introduced by fixing the error in Fig 4.
- Fig S9 added
- Fig S10 added
- Number of each figure and table adjusted for inclusions of new figures/tables
